# Current developments in the diagnosis and treatment of giant cell arteritis

**DOI:** 10.3389/fmed.2022.1066503

**Published:** 2022-12-13

**Authors:** Denes Szekeres, Bayan Al Othman

**Affiliations:** ^1^School of Medicine and Dentistry, University of Rochester Medical Center, Rochester, NY, United States; ^2^Department of Ophthalmology, University of Rochester Medical Center, Rochester, NY, United States

**Keywords:** giant cell (temporal) arteritis, color Doppler ultrasonography (CDUS), biologic therapeutics, clinical trials, diagnostics - clinical characteristics

## Abstract

Giant cell arteritis is the most common vasculitis in adults above 50 years old. The disease is characterized by granulomatous inflammation of medium and large arteries, particularly the temporal artery, and is associated acutely with headache, claudication, and visual disturbances. Diagnosis of the disease is often complicated by its protean presentation and lack of consistently reliable testing. The utility of color doppler ultrasound at the point-of-care and FDG-PET in longitudinal evaluation remain under continued investigation. Novel techniques for risk assessment with Halo scoring and stratification through axillary vessel ultrasound are becoming commonplace. Moreover, the recent introduction of the biologic tocilizumab marks a paradigm shift toward using glucocorticoid-sparing strategies as the primary treatment modality. Notwithstanding these developments, patients continue to have substantial rates of relapse and biologic agents have their own side effect profile. Trials are underway to answer questions about optimal diagnostic modality, regiment choice, and duration.

## 1 Introduction

Large vessel vasculitis (LVV) refers to a spectrum of diseases unified by granulomatous inflammation of the aorta and its major branches. Takayasu arteritis and giant cell arteritis (GCA) are the major entities of LVV, differing in primarily in their age of onset. The focus of this review will be GCA, the most common vasculitis in adults above 50 years old. While patients may present classically with headache, jaw claudication and visual disturbances in the setting of other constitutional symptoms, there is a wide spectrum of disease (1). Disease flares may cause permanent vision loss, cerebral ischemia or aortic aneurysms if not treated promptly with corticosteroids. Often, patients will require other adjunctive therapeutics to prevent relapse or treat steroid-refractory disease. Since the first histological description of GCA in the early 20th century, (2) there have been numerous developments in elucidating its pathogenesis and optimizing its management. The present paper will review the disease with mention of diagnostic advancements, shifts in treatment strategies, and several landmark trials exploring novel therapeutics.

## 2 Pathophysiology

**FIGURE 1:**
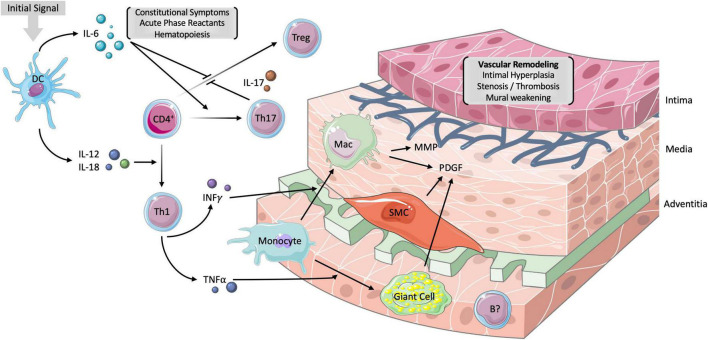
Vascular pathogenesis of GCA. The inciting factor for the development of GCA is unknown but though to involve the activation of resident dendritic cells via their toll-like receptors. The subsequent release of interleukin IL-6 and IL-12 promotes the differentiation of CD4^+^ T-helper cells (Th) into Th17 and Th1 cells, respectively. Th17 cells release IL-17 which is responsible for inhibiting T-regulatory (Treg) cells, stimulating hepatic acute phase reactants (APRs) and monocytes. Th1 cells release mediators of chronic inflammation, including interferon gamma (IFNγ) and tumor necrosis factor alpha (TNFα). While the former stimulates monocyte differentiation into macrophages, the latter influences the formation of multinucleated giant cells. The net of effect of macrophages, smooth muscle cells (SMCs) and giant cells is the release of platelet-derived growth factor (PDGF). PDGF is responsible, in part, for the intimal hyperplasia, stenosis, and thrombosis of large vessels in GCA. Matrix metalloproteinases (MMP) are derived from macrophages and result in the degradation of the tunica media. The resulting vascular remodeling underlies aneurysms in GCA. B cells have no established role in GCA but have been found in the adventitia of arterial biopsies and are implicated in certain chemokine axes. IL-6 is responsible for the constitutional symptoms such as fever and malaise as well as the production of APRs. DC, dendritic cell; CD4^+^, CD4^+^ T-helper cell; Mac, macrophages; IL, interleukin. Parts of the figure were drawn by using pictures from Servier Medical Art. Servier Medical Art by Servier is licensed under a Creative Commons Attribution 3.0 Unported License (https://creativecommons.org/licenses/by/3.0/).

The granulomatous inflammation of the medium- and large-sized vessels arising from the aortic arch is mediated by a slew of cellular and humoral immune components (Figure 1). The inciting factor for development of GCA is unknown but thought to be virus-related. Resident dendritic cells were shown to be the first immune elements that are activated via their toll like receptors (TLRs) (3). Mature dendritic cells release a variety of chemokines that trigger the recruitment and differentiation of various members of the CD4^+^ T cell lineage.

The release of IL-1β, IL-6, and IL-23 from dendritic cells induce the differentiation of CD4^+^ T cells into T-helper 17 (Th17) cells. The Th17 subtype, and their derivative cytokines IL-17, IL-22, and GM-CSF, play a vital role in initiating the pro-inflammatory response. Th17 cells stimulate the hepatic production of acute phase reactants (APRs) and other immune cells such as monocytes (4). The dendritic and Th17 cell pools are also responsible for the downregulation of T regulatory (Treg) cells through IL-6 and IL-17, respectively. The blunting of the typical anti-inflammatory balance is, at least partially, responsible for the chronic nature of GCA (5).

Dendritic cells additionally induce the differentiation of CD4^+^ T cells into Th1 cells through IL-12 and IL-18. Th1 cells tend to release mediators of chronic inflammation, including interferon gamma (IFN-γ) and tumor necrosis factor alpha (TNF-α). IFN-γ is involved in the activation of vascular smooth muscle cells and the recruitment of monocytes and their differentiation into macrophages. Histiocytes subsequently form the eponymous multinucleated giant cell under the influence of TNF-α (6). Notably, while the Th17 cells appear to be modulated by glucocorticoid therapy, the Th1 subtype remains active in chronic disease (7).

While B cells play a less significant role in the pathogenesis of GCA, there have been implications of B cell pool dysregulation in recently diagnosed patients (8). A 2019 histological examination of 9 aortic biopsies in patients with GCA found adventitial B cell infiltrate organizing into a lymphoid pattern typical of large vessel vasculitides such as Takayasu arteritis (9). Two chemokine axes, CXCL9-CXCR3 and CXCL13-CXCR5, have been implicated in the recruitment and organization of B cells in GCA, though further work should elucidate its role as a therapeutic target (10).

Macrophages were shown to play a key role in intimal hyperplasia and angiogenesis through the release of platelet derived growth factor (PDGF) and matrix metalloproteinases (MMPs) (11–13). Vascular stenosis and thrombosis from intimal hyperplasia is responsible for the jaw claudication and ocular manifestations of GCA. MMPs degrade the media and are responsible for vessel aneurysm. Cytokines, particularly IL-6, remain the backbone of the systemic inflammatory reaction and are responsible for the constitutional signs such as fever, malaise, and myalgias. The culmination of these inflammatory mediators in GCA alludes to the many potential targets for novel steroid-sparing therapy.

## 3 Clinical presentation

The spectrum of symptoms in patients with GCA are a sequela of vascular occlusion and thus prompt vascular, ophthalmologic, rheumatologic, and neurologic workups. The GCA disease spectrum encompasses three broad phenotypes: Cranial GCA (C-GCA), Large Vessel GCA (LV-GCA), and mixed. C-GCA is associated with the prototypical symptoms of GCA including headache, temporal artery abnormalities and visual disturbances. LV-GCA includes the development of aneurysms or arterial stenoses and presents with limb claudication and aortitis, alongside the traditional symptoms (14). While large vessel involvement occurs in less than half of patients with any GCA, it is associated with increased mortality (15, 16). The mixed phenotype includes features of both C-GCA and LV-GCA and may represent nearly 80% of GCA cases (17).

There appear to be additional associations with polymyalgia rheumatica, another common inflammatory disease with similar epidemiology, pathophysiology, and presentation. A recent meta-analysis of 566 patients found that over 25% of patients with polymyalgia rheumatica (PMR) may present with signs of subclinical GCA, particularly increased aortic uptake in PET scanning (18). Best practice management for these subtypes remains an area of continued investigation (19).

Certain constitutional symptoms are present in most patients with 50% of patients experiencing a low-grade fever, though in some patients the only presenting symptoms may be myalgias. Beyond fever, headache with scalp tenderness, fatigue, facial pain, and weight loss have all been associated with GCA. Notably, one meta-analysis found that the presence of temporal headache did not confer a significantly higher likelihood ratio for diagnosis of GCA. However, other vaso-occlusive signs such as jaw and limb claudication were more sensitive for diagnosis of GCA (20).

Temporal artery abnormalities may present as nodular, tortuous swellings of the vessel with possible loss of pulsation. These findings are secondary to the intimal hyperplasia and sclerosis from chronic inflammation and macrophage-derived PDGF and MMPs (13). A 2021 meta-analysis collected data from 68 studies and approximately 4,000 biopsy-confirmed unique cases of GCA. The authors suggested that any temporal regional abnormality or temporal arterial tenderness or pulselessness doubled the odds, at minimum, for a positive biopsy (21).

Data from a large population-based cohort found that visual changes occur in around 20% of patients and progressing to vision loss occurs in less than 5% (22). Commonly, patients will report a transient, painless monocular vision loss (i.e., amaurosis fugax), though it may be painful in up to 10% of patients (23). Vision loss in GCA is often secondary to arteritic anterior ischemic optic neuropathy (AAION) due to occlusion of the short posterior ciliary arteries that supply the choroid and optic disk. This phenomenon appears as “chalk white” optic disk edema with possible hemorrhage and cotton-wool spots on fundoscopic examination. Less commonly, GCA-associated vision loss may be due to posterior ischemic optic neuropathy or central retinal artery occlusion. Rarely, patients will present with diplopia due to ischemia of extraocular muscles or visual hallucinations, as described in previous literature (23–25).

### 3.1 Other associations

GCA symptomology may often be vague and variable. The malaise, headache, fever, and elevated CRP in conjunction with rare reports of dry cough, have generated some diagnostic confusion with COVID-19. Associated diagnostic delay has been suggested to be responsible for increase morbidity from GCA in a single-center fast-track program (26). A 2021 systematic literature review of several cohorts compared the clinical presentation of GCA and COVID-19 and identified key distinguishing features. Jaw claudication and visual loss were rarely reported in COVID-19 cases while lymphopenia appeared nearly exclusively in GCA (27). Interaction of the two disease processes, particularly due to the upregulation of IL-6 and IL-7 in both conditions, has the potential to produce serious adverse outcomes, as described in two case studies with GCA-associated visual loss (28, 29). How, if at all, management is adjusted based on COVID remains an area of investigation.

### 3.2 Relapse

Relapse during or after glucocorticoid therapy has been reported in over half of patients and up to 21% experience multiple relapses. One study found that relapse appeared independent of glucocorticoid dosage and often appeared while undergoing treatment (30). Other risk factors for relapse are less well established, with a recent study showing that LV-GCA, a negative TAB, primarily musculoskeletal symptoms, and female gender were all associated with an increased risk (31). Another recent trial found that higher platelet count and a glucocorticoid-induced transcript 1 polymorphism reduced the risk of relapse (NCT01400464) (32). While relapse symptoms are often milder – reporting as being headaches, PMR-like symptoms, or claudication – patients would nevertheless benefit from treatments with sustained remission (33, 34).

## 4 Diagnostics

Even before obtaining confirmatory diagnostic testing, immediate treatment with corticosteroids and tocilizumab is recommended in cases with high suspicion of GCA. Criteria for the diagnosis of GCA were originally set forth in 1990 by the American College of Rheumatology (ACR; Table 1). Accordingly, patients are deemed to have GCA and are recommended TAB if they meet three or more of the five criteria, with a sensitivity of 93.5% and specificity 91.2% (35). One major pitfall of the criteria arises in cases of very low or high pretest probability; for example, if a patient presents with a new headache over the age of 50 with elevated ESR, they are recommended a TAB. Whereas these protean symptoms may be sequelae of malignancy, infection, or other autoimmune conditions, rather than GCA. A recent paper proposed a revised set of criteria (rACR) to avoid temporal artery biopsy in cases such as above or in those with cardinal symptomology. The rACR stratifies criteria into two domains, one encompassing the cardinal and the other the protean signs and symptoms, for a total of nine points (Table 2). In a review of the criteria 100% of patients scoring five or more had a positive biopsy and thus could possibly avoid biopsy. A score of three or more detected 91% of positive cases, whether or not it is acceptable to miss one to two cases to avoid biopsy is debated (36, 37).

**TABLE 1:** 1990 ACR Guidelines for GCA.

Score	Criterion
1	Age at disease onset greater than 50 years
1	New headache
1	Temporal artery abnormality ^♢^
1	Erythrocyte sedimentation rate greater than 50 mm/hr
1	Abnormal temporal artery biopsy

A patient is deemed to have GCA and are recommended to have a TAB if they meet three or more of these criteria.

^♢^Including tenderness to palpation or decreased pulsation.

**TABLE 2:** rACR Guidelines for GCA.

Score	
**Entry Criterion**	

–	Age at disease onset greater than 50 years
–	Absence of exclusion criteria^♢^

**Domain I**	

1	New onset localized headache
1	Sudden onset of visual disturbances
2	Polymyalgia Rheumatica (PMR)
1	Jaw Claudication
2	Abnormal temporal artery on physical exam

**Domain II**	

1	Unexplained fever or anemia
1	ESR greater than 50 mm/hr
2	Compatible pathology^∇^ on biopsy

Expanded set of criteria across two domains of presentation. Patients with three points out of the eleven total are diagnosed with GCA.

^♢^Including tenderness to palpation or decreased pulsation.

^∇^Fibrinoid necrosis with perivascular leukocyte invasion and granulomas.

The two main governing bodies, the ACR and EULAR, recently released joint guidelines for the classification of GCA (Table 3). These guidelines are applied after the diagnosis of a medium- or large-vessel vasculitis is established to further classify the presentation as GCA. Importantly, these are not aimed to be used as initial diagnostic criteria. Analysis of the 2022 criteria found a sensitivity of 87% and specificity of 95%, with superior sensitivity when compared to the 1990 ACR criteria (38).

**TABLE 3:** 2022 ACR/EULAR Classification Guidelines for GCA.

Score	
**Absolute requirement**	

–	Age at disease onset greater than 50 years

**Additional clinical criteria**	

2	Morning stiffness in shoulders or neck
3	Sudden onset visual loss
2	Jaw or tongue claudication
2	New temporal headache
2	Scalp tenderness
2	Temporal artery abnormality ^♢^

**Laboratory, Imaging, and Biopsy Criteria**	

3	Maximum ESR greater than 50 mm/hr or maximum CRP greater than 10 mg/L
5	Positive temporal artery biopsy or positive halo sign on temporal artery ultrasound
2	Bilateral axillary involvement ^∇^
2	FDG-PET activity throughout aorta

These criteria classify medium- or large-vessel vasculitis as GCA after excluding other etiologies. A sum of scores greater than or equal to 6 is deemed positive for GCA.

^♢^Including tenderness to palpation, cord-like appearance, or decreased pulsation.

^∇^Angiography showing luminal stenosis, increased uptake on FDG-PET, halo sign on ultrasound.

### 4.1 Temporal artery biopsy

TAB should be performed as soon as possible after beginning glucocorticoids and the ACR continues to recommend a long segment (>1 cm), unilateral biopsy in conjunction with clinical evaluation as the gold-standard for diagnosis. A retrospective cohort showed that biopsy results were positive in 78% of clinically-diagnosed GCA that started treatment within two weeks of TAB. A delay of over four weeks showed TAB-positivity in only 40% of patients, suggesting normalization of histologic findings (39). However, due to the small sample size of 78 patients with only five receiving TAB after four weeks, extrapolation to the broader clinical setting may be less robust. Nonetheless, a meta-analysis of 3,092 patients revealed that TAB had a pooled 77% sensitivity with a decreasing trend in positive biopsies, on par with other diagnostic testing (40).

Examination of temporal artery biopsies with hematoxylin and eosin staining typically shows panarterial lymphocytic infiltrates with granulomas. The evaluation may also reveal hyperplasia and fragmentation of the elastic laminae with minimal neutrophil invasion. Elastic van Gieson may reveal disruption of the internal elastic lamina and, while used for repeat biopsies, is not routinely recommended by the ACR (41). Skip lesions have been reported in roughly 10% of cases and raise concern for missed diagnoses, hence, the whole clinical picture and additional diagnostics remain important for thorough workup (42).

There are plethora patterns of GCA beyond the classic histological changes described above, which underlies the variability in disease presentation (43). One study proposed a model of sequential angioinvasion, beginning with adventitial involvement and ending with a panarterial inflammatory infiltrate. However, aside from an association of severe cranial symptoms with a panarterial pattern, the authors found few prognostic indicators based on histology alone (42).

### 4.2 Color Doppler ultrasound

Color Doppler Ultrasound (CDUS) is an ultrasonography technique that assesses directionality of blood flow. CDUS was first shown to be able to diagnose GCA in certain high pre-test probability cases in 1997 (44). Of particular importance is the ability to simultaneously image other cranial rami and large arteries including the axillary and subclavian, without added invasive testing. A meta-analysis of 43 individual studies found that CDUS has a specificity of 96% and sensitivity of 77% for GCA (45). The evaluation of ultrasound’s role in patients suspected of having extracranial and cranial giant cell arteritis or EUREKA study was a recent multicenter cohort study. Researchers demonstrated comparable specificities and sensitivities to prior work and showed that positive CDUS findings independently conferred a greater odds ratio for GCA diagnosis at six months than TAB alone (46). A separate multicenter prospective cohort study, the role of ultrasound compared to biopsy of temporal arteries in the diagnosis and treatment of giant cell arteritis, or TABUL, suggested that CDUS has superior sensitivity though inferior specificity compared to TAB (47). Collectively, these findings suggest CDUS may soon be considered the most appropriate first line test.

The prototypical finding in GCA is a dilated superficial temporal artery with a non-compressible, hypoechoic “halo” in the vessel wall, reflecting panarterial inflammation, thickening, and edema (44, 48). However, the incompressible halo sign is not pathognomonic for GCA. Many ultrasonographic features are shared among other ANCA-associated vasculitides, amyloidosis, and atherosclerosis, often complicating diagnosis in uncertain cases (49). Concerns were raised regarding poor inter-rater reliability described in one study (47). The implementation of recent training programs has shown good reliability with up to 96% interobserver agreement (48, 50, 51).

The prognostic and longitudinal utility of CDUS is still under investigation. The Halo Score is a recent development by *van der Geest* and colleagues and is predicated on the counting of halos in several temporal and axillary artery segments. While the sensitivity and specificity of this test alone was not superior to standard US workup, a high Halo Scores accurately identified patients at considerable risk for vision loss (52). Future work may explore the possibility of tailoring patients’ glucocorticoid dosing schedule based on such scoring.

Joint CDUS and TAB “fast-track pathways” are increasingly used by institutions (53–56). These programs employ a multidisciplinary team and structured algorithms to rapidly evaluate, diagnose, and define treatments for patients with suspected GCA. One study found significant reduction in vision loss due to faster time to diagnosis and initiation of treatment but no change in rates of relapse (55). The TABUL study demonstrated that ultrasound alone may provide comparable diagnostic accuracy with a significant reduction in cost and a theoretical 43% reduction in biopsies. Notably, the authors found that both ultrasonographers and pathologists had moderate interrater agreement (47). How, and if for all patients, fast-track pathways will continue utilizing TAB as a diagnostic standard is still under debate.

The use of CDUS to monitor disease progression is less well-documented. A 2018 systematic review found that the halo sign resolves in most patients undergoing adequate treatment, though no other reliable prognostic features were identified (45). The optimal use of CDUS remains a subject of investigation with recent literature examining the role of axillary (57, 58) and extended ultrasonographic evaluation in prognosis and disease monitoring (59, 60). Results of a recent study suggest that limited CDUS of the axillary arteries misses 4% of patients with LV-GCA identified by an extended exam (including carotid, vertebral, subclavian, and axillary arteries). Furthermore, in this study population, 9% of patients with LV-GCA had only vertebral artery involvement (59). Such extended examination requires advanced equipment and training that may ultimately be worthwhile for monitoring disease progression without the need for contrast agents used in other modalities. The advent of higher resolution probes may impact specificity of CDUS and its integration into practice is currently under investigation (NCT04204512).

### 4.3 FDG-Positron emission tomography

2-[Fluorine-18]-fluoro-2-deoxyglucose positron emission tomography (FDG-PET) is an imaging technique grounded in measuring metabolic activity and traditionally used for the diagnosis, staging and monitoring of malignancies. The modality has been shown to be applicable for hypermetabolic lesions such as GCA and other chronic inflammatory disorders with active cellular infiltrate (61). However, it is not considered a first-line diagnostic modality due its cost and radiation exposure. FDG-PET was first employed in tracking the involvement of large arteries in LV-GCA. Recent advancements in imaging technology permit the spatial resolution of the smaller cranial vessel involvement in C-GCA and distinguish lesions from the high background cranial uptake of FDG (62). Results from the Giant Cell Arteritis and PET Scan (GAPS) study showed that FDG-PET of the head and chest has a negative predicative value of 98%, though up to 20% of patients have additional incidental findings (NCT02771483) (63). A 2022 trial found that the combined use of cranial and extracranial FDG-PET decreased specificity and positive predictive value but increased sensitivity and negative predictive value in diagnosing GCA (NCT05246540) (64). The use of combined FDG-PET and magnetic resonance imaging (MRI) with angiography (MRA) has also been explored. While MRI alone can resolve GCA-associated intimal hyperplasia and other inflammatory changes (65), combined FDG-PET/MRI provides an additional lens to evaluate the underlying biochemical mechanisms (66, 67). How this will change standard imaging is under evaluation and it’s use as the sole diagnostic test is under scrutiny (NCT04204876 & NCT05000138). Considering the cost of these modalities, FDG-PET may be less practical than point-of-care CDUS.

The role of FDG-PET in prognosis and longitudinal evaluation is similarly unclear. FDG uptake did not appear to distinguish patients with active disease and those in remission (68) and appeared to normalize within three days of beginning glucocorticoid therapy (69). In LV-GCA, increased aortic FDG uptake was shown to be associated with an increased risk of thoracic aneurysm (70, 71). To date, no other correlations with disease patterns have been elucidated and there remains a paucity of research on the clinical impact of FDG-PET results.

The focus of current research is the discovery of novel radiotracers that may have improved specificity for GCA. A recent trial was started comparing the use of Ga-DOTATATE to FDG for the detection of inflammation and its potential to correlate with disease activity in patients receiving glucocorticoids (NCT03812302). The use of a somatostatin receptor tracer is also under investigation (NCT04071691). While many other tracers are currently in trial for their use in monitoring cancers, T cell- (72) and macrophage-specific (73, 74) tracers have been shown to identify areas of vascular inflammation and could be extrapolated to GCA. These studies are reviewed in detail elsewhere (75).

### 4.4 Magnetic resonance imaging

MRI is a high-resolution imaging modality that has been shown to be effective in evaluating inflammation of cranial vessels. Imaging may show evidence of luminal stenosis, vessel dilatation or aneurysms. Compared to TAB, MRA was 93% sensitive and 81% specific for GCA (45). The ACR recommends the use of MRA for the diagnosis of GCA if biopsy or CDUS is inconclusive (41). Interestingly, a 2022 study demonstrated that while CDUS, MRA, and retinal angiography were independently accurate, a combination of MRA followed by CDUS if inconclusive was 100% sensitive, specific, and accurate (76). These findings support EULAR guidelines which recommend a multi-modality diagnostic approach. Additional studies are needed to compare FDG-PET with MRA.

Disease monitoring after initial presentation is also recommended based on institutional availability to evaluate the extent of large vessel aneurysms and stenoses. Imaging frequency and modality should be determined by joint patient physician decision-making. Compared to CDUS, a small study found that MRA did not have significant differences in sensitivity and specificity in the diagnosis of GCA, when compared to TAB (77). A more recent cross-sectional study found that CDUS was more sensitive in detecting vasculitic changes in large vessels compared to MRA (78). Given the higher cost of MRA and the exposure to contrast, CDUS is often a more appropriate test.

### 4.5 Conventional angiography

Computed tomography angiography (CTA) has been used in the historical evaluation of large vessel involvement in GCA and often shows wall thickening with a double ring of contrast enhancement. A small case-control study showed that CTA was able to resolve superficial temporal artery abnormalities such as perivascular contrast enhancement and blurring of vessel walls (79). Several studies (80, 81) suggest that PET/CT provides superior sensitivity over CTA alone. This is reflected in the EULAR guidelines, which do not routinely recommend that the diagnosis of GCA or evaluation of LVV hinge on CTA (82).

### 4.6 Laboratory markers

The inflammatory milieu in generalized inflammation stimulates the hepatocellular production of C-reactive protein (CRP). While CRP is a direct marker of inflammation, erythrocyte sedimentation rate (ESR) is a surrogate marker, reflecting the increase in fibrinogen that may occur secondary to many conditions. Measurement of these markers is standard in the workup of GCA, and both are often markedly elevated in patients with acute disease. ESR above 100 mm/hr was found to be associated with a 3-fold increase in likelihood of GCA, whereas ESR below 40 mm/hr or a CRP below 2.5 mg/dl nearly halved the likelihood of GCA (20).

### 4.7 Diagnostic guidelines

Both the ACR and EULAR have recently updated their guidelines to reflect the advancements in diagnostic imaging. While largely similar, there are some differences in the diagnostic guidelines set forth by the ACR and the EULAR. Notably, the ACR guidelines continue to endorse a TAB over temporal ultrasound owing largely to differences in ultrasonographic training. While they recommend adjunctive large vessel imaging after confirmation by biopsy, angiographic imaging alone is not deemed sufficient for initial diagnosis (41). Contrarily, the EULAR cite a strong level of evidence for diagnosis without biopsy in cases of positive cranial MRA or temporal and axillary ultrasound (82). Longitudinal imaging, while evidenced to have value in monitoring structural damage, is not routinely recommended by the EULAR. Instead, personal preference and cost-benefit analysis should drive clinical decision-making when evaluating disease flares. The ACR recommends that some form of longitudinal clinical monitoring be done, whether it be clinical examination, laboratory evaluation or imaging. In light of the recent 2022 joint ACR/EULAR classification criteria, unified diagnostic guidelines may be on the horizon.

## 5 Management

### 5.1 Glucocorticoids

Glucocorticoids have been vital for the acute and chronic treatment of GCA. Through several mechanisms, including inhibition of the Th17 cell pool, glucocorticoids modulate inflammation and effectively reduce the risk of vision loss. Oral glucocorticoids are often initiated at 1 mg/kg/day with higher dosing for patients with severe ophthalmologic symptoms. The British Society of Rheumatology (BSR) recommends pulsed intravenous (IV) administration of up to five days of 1,000 mg methylprednisolone for patients with high-risk features (83). However there are no randomized controlled trials (RCTs) comparing outcomes of either route, thus clinical decision-making is largely consensus-based (84). The side effect profile of IV glucocorticoids, especially in the elderly populations where GCA is prevalent, should also be taken into consideration when selecting initial treatment.

After initiation, glucocorticoids are generally tapered over the course of a year, although there are differences in published guidelines. In the United States tapering is often six to eight months while the EULAR recommends 18 to 24 months of tapering. A European trial is currently underway comparing rates of remission and side effects for 28- and 52-week tapering regiments (NCT04012905).

### 5.2 Glucocorticoid-sparing therapies

Patients will often restart courses of glucocorticoids to manage flares, substantially increasing their cumulative exposure. While longer regiments effectively reduce serious GCA-related adverse events, repeated glucocorticoid therapy harbors its own set of serious side effects. Often cited side effects of glucocorticoids include newly diagnosed hypertension, diabetes mellitus, as well as osteonecrosis, increased rates of infections, and cataracts. One case-control study in patients with GCA found that higher cumulative dose of glucocorticoids (30 versus 5 mg/day) was associated with a nearly five-fold increased risk of diabetes mellitus, a two-fold increased risk of osteoporosis and two-fold increased risk in all-cause mortality (85). A larger study based on data from US and UK databases concluded that for each gram of cumulative glucocorticoid exposure, there is a three to eight percent increase in risk of any steroid-related adverse event (86).

Diverse classes of adjunctive therapies have been explored since the initial treatment of GCA with glucocorticoid monotherapy decades years ago (Table 4). Many of these therapeutics are currently under laboratory investigation and, to date, methotrexate and tocilizumab are the only FDA-approved treatments in the United States. Guidelines for treatment of the initial disease and subsequent flares are shifting toward prioritizing the newly licensed glucocorticoid-sparing therapy tocilizumab. The follow sections review the noteworthy investigational drugs by their therapeutic class.

**TABLE 4:** Clinical trials for GCA treatment.

Pathway	Drug target	Agent	Class	Trials
**Cytokine signaling**				
	IL-1	Anakinra	Recombinant IL-1R antagonist	NCT02902731
	IL-6	Tocilizumab	mAb	NCT01791153, NCT03202368, NCT04239196, NCT03745586, NCT05479448, and NCT05045001
	IL-6	Sirukumab	mAb	NCT02531633
	IL-6	Sarilumab	mAb	NCT03600805
	IL-17	Secukinumab	mAb	NCT03765788, NCT05380453, and NCT04930094
	IL-12/IL-23	Ustekinumab	mAb	NCT03711448
	IL-23	Guselkumab	mAb	NCT04633447
	TNFα	Infliximab	mAb	NCT00076726 and NCT05168475
	TNFα	Etanercept	mAb	NCT00524381 and NCT05168475
	TNFα	Adalimumab	mAb	NCT00305539 and NCT05168475
	GM-CSFRα	Mavrilimumab	mAb	NCT03827018
**JAK-STAT signaling**				
	JAK1/JAK2	Barcitinib	Small molecule	NCT03026504
	JAK1	Upadacitinib	Small molecule	NCT03725202
**T-lymphocyte**				
	CTLA4 Analog	Abatacept	Selective costimulatory modulator	NCT04474847

Multiple agents have been explored for the treatment of GCA, with particular interest in the cytokine signaling pathways. Drug targets, classes and respective trials are reviewed. IL, interleukin; TNF, tumor necrosis factor; GM-CSF, granulocyte-macrophage colony stimulating factor; JAK-STAT, janus kinase signal transducer and activator of transcription; CTLA, cytotoxic T-lymphocyte-associated protein; mAb, monoclonal antibody.

#### 5.2.1 Non-biologic adjuncts

##### 5.2.1.1 Methotrexate

Methotrexate is a dihydrofolate reductase anti-metabolite used to treat a variety of malignancies due to its antagonism of DNA synthesis. Its mechanism in treating autoimmune disorders involves inhibiting the breakdown of adenosine and preventing the activation of T- and B-cells. Methotrexate is the most common non-biologic agent used in addition to glucocorticoids for the management of GCA (87). To date, only three RCTs have been performed with results showing either no difference in rates of relapse (88, 89) or reduction in relapse from 84 to 45% (90). A pooled meta-analysis suggested a 35% reduction in risk of first relapse with significant reduction in total glucocorticoid exposure versus placebo. Whether or not the glucocorticoid-sparing effect of methotrexate outweighs it side effect profile remains unclear from this study (91).

The ACR currently recommends the use of methotrexate based on clinician experience and patient preference (41). How methotrexate will continue to play a role in GCA management is under debate, principally due to the marked efficacy of tocilizumab (92). One advantage of methotrexate is that it is a small molecule chemical and trends significantly cheaper than contemporary biologics. A 2020 RCT is evaluating efficacy of a 12-month treatment of methotrexate versus tocilizumab in 200 patients (NCT03892785). The authors hypothesize that rates of remission will be comparable, resulting in superior cost efficiency. Other non-biologic adjuncts such as azathioprine (93), cyclosporine A (94), and dapsone (95) have been studied but yielded, at most, modest results with strikingly poor side effect profiles.

##### 5.2.1.2 Leflunomide

Leflunomide, with its active metabolite teriflunomide, inhibits dihydroorotate dehydrogenase, a mitochondrial enzyme involved in pyrimidine synthesis. It has been shown as an effective agent in RA, Takayasu arteritis, and PMR (96, 97). Its use in clinical practice is based on results from smaller case reports and open-label studies. Several case series’ showed efficacy and good tolerability with steroid-sparing effect in patients with GCA (98, 99). A 2018 observational study demonstrated significant reduction in relapse compared to the glucocorticoid group with a significantly lower cumulative steroid dose (100). Compared to methotrexate, leflunomide appeared to induce remission earlier, particularly in patients requiring higher doses of prednisolone initially (101). To date, there have been no randomized controlled trials comparing leflunomide with standard therapies.

#### 5.2.2 Interleukin pathway inhibitors

##### 5.2.2.1 Interleukin-1

Anakinra, an IL-1 receptor antagonist, showed efficacy in a case series of six patients. Notably, the authors found disappearance of aortitis in one patient and reduction in FDG vascular uptake in three. Four patients achieved steroid-free remission by median 56 months (102). Though the results appear promising, data from an ongoing trial, the Giant Cell Arteritis and Anakinra Trial (GiAnT), may clarify its efficiency compared to placebo (NCT02902731).

##### 5.2.2.2 Interleukin-6

IL-6 is the predominant cytokine in the pathogenesis of and was found to be consistently elevated in GCA. Tocilizumab, sold under the trade name Actemra^®^, is a humanized anti-IL-6 receptor monoclonal antibody first used for the treatment of multicentric Castleman disease in 2009 (103). The Giant-Cell Arteritis Actemra (GiACTA) trial is a phase 3 RCT that showed a weekly or biweekly dose of 162 mg tocilizumab with prednisone provided superior rates of remission and longer flare-free intervals than patients solely on prednisone. Adverse events occurred in about 15% of patients receiving tocilizumab but over 22% of patients on placebo and prednisone taper (104). Even with promising results from trials, up to 40% of patients relapsed after cessation of treatment with tocilizumab. Newer research appears to suggest that long-term therapy with tocilizumab may be appropriate. The incidence of adverse events is comparable between treatment regiments greater than or less than a year, with clinical improvement in 90-100% of patients by 24 months (105, 106). Both EULAR (107) and the 2021 ACR (41) guidelines have shifted to recommend tocilizumab with a glucocorticoid taper for both the initial treatment of GCA and management of subsequent flares. Studies of different dosing schedules and routes (108) as well as long-term safety profiles (NCT03202368) are currently underway.

IL-6 is key player in the healthy immune response against infection and blockade of this system is responsible for the increased risk of infection with use of tocilizumab (10% patients per year) (105). As with other immune modulating agents, screening for tuberculosis is recommended prior to beginning treatment. Tocilizumab was also shown to increase the risk for bowel perforation and has been documented to increase lipids in some patients. Trimonthly laboratory monitoring for neutropenia (occurring in about 4% of patients), thrombocytopenia, and hyperlipidemia as well as liver function testing is recommended during treatment.

Sirukumab is another humanized anti-IL-6 monoclonal antibody that entered phase three trial in 2015 (NCT02531633). While results are limited due to early study termination by the sponsoring agency, sirukumab with a prednisone taper was found to reduce number of flares compared to placebo with taper. There were no reports of bowel perforation, but the rates of infection and laboratory abnormalities were consistent with those found in the tocilizumab trial. A related biologic, sarilumab, was under investigation until its suspension in April 2020 due to COVID-19 (NCT03600805).

##### 5.2.2.3 Interleukin-17

IL-17 from Th17 cells is responsible for part of the GCA inflammatory response. While glucocorticoids have already been shown to inhibit the Th17 axis, targeting with biologics may provide additional benefits. Secukinumab is a humanized anti-IL-17A monoclonal IgG antibody, sold under the brand name Cosentyx^®^, that is currently FDA-approved for the treatment of plaque psoriasis. A phase two trial showed efficacy and an acceptable safety profile of secukinumab versus placebo (NCT03765788). Two recent phase 3 trials are underway and will compare the use of secukinumab with a prednisone taper versus a placebo with taper (NCT05380453 & NCT04930094).

##### 5.2.2.4 Interleukin-12 & Interleukin-23

The IL-12 and IL-23 pathways are the targets for the monoclonal antibody ustekinumab, which may modulate the Th1 and Th17 response simultaneously. A smaller study found that ustekinumab induced complete remission and successfully lowered the total glucocorticoid dose for 14 patients (109). A follow-up open-label study of 13 patients showed poor outcomes with very high rates of relapse (110). In light of these mixed findings, a newer phase two open-label study might better show the efficacy of ustekinumab (NCT03711448). Janssen Pharmaceuticals is currently comparing the use of guselkumab, an IL-23 specific receptor antagonist against placebo in 60 patients (NCT04633447).

#### 5.2.3 T-Llymphocyte modulators

T cell activation requires a CD28-mediated costimulatory signal from antigen presenting cells (APCs). Several trials have explored blocking T cell activation with abatacept, a biologic CTLA4 analog that binds B7 protein on APCs. The first placebo-controlled study showed longer duration of remission but had similar rates of adverse effects compared to prednisone alone (111). Its clinical use is still under investigation with a phase three trial currently underway (NCT04474847). Notably, there was a recent 28-patient study directly comparing the efficacy of abatacept against tocilizumab. In the study cohort, tocilizumab appeared to have superior rates of remission and reduced the cumulative dose of steroids compared to abatacept (112).

#### 5.2.4 TNFα inhibitors

TNFα has been identified in the arteries of patients with GCA. Single case studies and case series’ have shown some promise in treatment of GCA (113–116). However, RCTs of infliximab (117), etanercept (118), and adalimumab (119) did not appear to significantly improve outcomes versus placebo and the risk for infections were noticeably higher.

#### 5.2.5 JAK/STAT pathway inhibitors

The Janus Kinase (JAK) and signal transduction activator of transcription (STAT) pathway induces DNA transcription from extracellular ligands such as cytokines. Both Th1 and Th17 have been linked to STAT proteins and thus the JAK/STAT pathway is thought to play a role in the inflammation of large-vessel vasculitides (120). Indeed, mouse models of vasculitis have shown that inhibition of the JAK/STAT pathway may blunt the production of inflammatory mediators and thus is a feasible target for therapeutics (121).

Baricitinib, sold under the trade name Olumiant^®^, is a small molecule inhibitor of JAK1 and JAK2 first licensed for the treatment of TNF antagonist-resistant rheumatoid arthritis in 2018. In 2022 it was also licensed for the treatment of alopecia areata and COVID-19. Recently published results from a pilot study of baricitinib (NCT03026504) showed good tolerability and durable glucocorticoid-free remission in 13 of 15 patients (122). Upadacitinib (Rinvoq^®^), another small molecule inhibitor approved for the treatment of rheumatoid arthritis, is currently under investigation for its use in GCA (NCT03725202).

Despite promising rates of remission, JAK/STAT inhibitors carry an increased risk of infection. As with other immunomodulating therapies, further research and cost-benefit analyses need to be done before its role in the treatment of GCA is solidified.

#### 5.2.6 GM-CSFRα inhibitors

GM-CSF was identified as a key component in the pathogenesis of GCA. Mavrilimumab, a humanized monoclonal antibody that targets the GM-CSF Receptor alpha chain, is a therapeutic agent that was first investigated as a treatment for rheumatoid arthritis in 2011 (123). A recent phase 2 trial showed lower rates of remission and longer time to flares compared to glucocorticoid taper in 42 patients (NCT03827018). While the study reported no serious adverse effects, further work needs to be done to assess long-term efficacy and compare it to tocilizumab and other investigational therapeutics (124).

### 5.3 Surgical interventions

Surgical interventions in GCA are primarily aimed at ameliorating vascular injuries to the aorta and its major branches. Aside from urgent intervention in cases of dissection or ischemia, the ACR recommends elective surgeries based on patient preference, healthcare team consensus, and during disease remission (41). Vessel stenosis can occur at any time during the disease course, with cases of critical limb ischemia requiring venous bypass grafting or endovascular repair (125, 126). Commonly, the bypassed or repaired vessel may fail either from anastomotic aneurysm or re-occlusion. A recent case series also demonstrated successful endovascular repair of intra-cranial vessels in patients with tocilizumab-resistant disease and stroke (127). Endovascular repair will likely continue to evolve and play a larger role in cases of GCA with severe angiopathy (128).

## 6 Discussion

Giant cell arteritis is a granulomatous inflammation of medium and large arteries and is the most common vasculitis in older adults. While it remains a diagnostic challenge, the use of ultrasound has now complemented the traditional temporal artery biopsy as standard workup, though supplementary testing including FDG-PET and MRI are used in certain scenarios. Advancements in diagnostics and the development of streamlined programs have benefited countless patients by reducing time to treatment and improving disease monitoring. Several trials continue to investigate the role of these modalities. One study is currently validating a diagnostic CDUS algorithm and is pending results (NCT02703922). One aims to answer how the diagnostic accuracy of CDUS and FDG-PET change with the onset of treatment (NCT03765424). Another prospective study is directly comparing the clinical use of common diagnostic modalities in GCA diagnosis (NCT05248906). Certainly, with new evidence, more concrete diagnostic algorithms will be implemented.

While there is much research underway for novel agents, there remains a debate about best practice with current standards of treatment. Does time of glucocorticoid taper impact rates of remission and risk of side effects? How do parameters for glucocorticoid taper change with the use of tocilizumab? Does tocilizumab benefit from glucocorticoid administration or is monotherapy sufficient? Does tocilizumab in combination with glucocorticoids reduce risk of AION (NCT04239196)? Where does methotrexate enter the equation and is this antiquated drug still relevant for treatment? Results from several trials in the coming years will hopefully answer some of these questions. The GCA treatment with ultra-short GC and tocilizumab (GUSTO) trial (NCT03745586) showed that a three-day course of high dose glucocorticoids had adequate rates of remission in 13 of 18 patients, comparable to the standard 24-week taper course (129). Another 30-patient open-label trial showed that 12 months of tocilizumab with initial two months prednisone taper was able to induce remission in 77% of patients by 12 months (130). Where along this spectrum is the optimum treatment and can we predict which patients will respond to glucocorticoids or tocilizumab (NCT05479448 & NCT05045001)?

Tocilizumab has shown promise in improving remission and glucocorticoid-associated complications, but the therapy is expensive, confounds common biomarkers for monitoring, and 50% may relapse after cessation of treatment. In the coming years, head-to-head comparisons of efficacy and safety between these anti-interleukin therapies may change best practice guidelines. Already, a RCT comparing the efficacy of the biologics rituximab, infliximab, and tocilizumab using a crossover design is underway in the United Kingdom (NCT05168475). The study is enrolling a broad patient population with diagnoses of any non-ANCA-associated vasculitides including polyarteritis nodosa, Takayasu arteritis, and GCA, among others. While it is the first trial to date directly comparing several biologic agents, the number of patients with GCA may be limited, lessening the power of the study and its extrapolation to GCA specifically.

Considering all these advancements GCA remains a chronic disease, patient choice and quality of life should still drive treatment decisions. Is there any way to determine who is at risk (NCT01241305 & NCT02967068) and are there any preventative measures that can improve patient outcomes? For those already undergoing treatment, can we give hydrocortisone (NCT042391960) or other ‘rescue’ therapies to improve quality of life?

## Author contributions

BA conceived the idea and scope of the review, reviewed and approved the manuscript. DS performed the literature review and wrote the manuscript. Both authors agreed to be accountable for the content of the work and contributed to the article and approved the submitted version.
